# Capsule colonoscopy increases uptake of colorectal cancer screening

**DOI:** 10.1186/1471-230X-12-80

**Published:** 2012-06-26

**Authors:** Stefan Groth, Horst Krause, Rainer Behrendt, Helge Hill, Michael Börner, Murat Bastürk, Nora Plathner, Friedrich Schütte, Ulrich Gauger, Jürgen Ferdinand Riemann, Lutz Altenhofen, Thomas Rösch

**Affiliations:** 1 Department of Interdisciplinary Endoscopy, University Hospital Hamburg-Eppendorf, Regional Hospital, Hamburg, Germany; 2 Private Gastroenterology Offices Rinteln, BKK 24 Medical Insurance, Obernkirchen, LebensBlicke Foundation for the Prevention of Colon Cancer, Ludwigshafen, Central Research Institute of Ambulatory Health Care, Berlin, Germany; 3 Department of Interdisciplinary Endoscopy, University Hospital Hamburg Eppendorf, Martinistr. 52, 20246, Hamburg, Germany

**Keywords:** Screening colonoscopy, Uptake, Capsule colonoscopy, Patient acceptability

## Abstract

**Background:**

Screening colonoscopy effectiveness is hampered by limited adherence by the general population. The present prospective study was performed to evaluate whether adding capsule colonoscopy to the endoscopic screening options increases uptake.

**Methods:**

Invitation letters were sent to 2150 persons above the age of 55 insured with a German medical insurance company in the area of Rinteln, Lower Saxony with a baseline spontaneous annual screening colonoscopy uptake of 1 %. Both capsule or conventional colonoscopy were offered. Interested persons were given information about the two screening options by four local gastroenterologists and examinations were then performed according to screenees’ final choice.

**Results:**

154 persons sought further information, and 34 and 90 underwent conventional and capsule colonoscopy, respectively. Colonoscopy uptake was thus increased by the invitation process by 60 % (1.6 % vs. 1 %; p = 0.075), while the option of capsule endoscopy led to a fourfold increase of screening uptake (4.2 % vs. 1 %, p < 0.001). Despite similar age distribution in both sex groups, uptake in men was significantly higher (5.6 % vs. 2.8 %, p = 002). However, overall adenoma yield was not different in both groups.

**Conclusions:**

The present study suggests that offering the option of capsule colonoscopy increases uptake of endoscopic colorectal cancer screening. However, capsule endoscopy sensitivity for adenoma detection needs to be improved.

## Background

There is adequate evidence that colorectal cancer (CRC) screening by various methods prolongs survival for those screened [1-3], and although this evidence is only indirect with regard to screening colonoscopy, the latter has been included in the CRC screening programmes of countries such as the USA and Germany. In Germany, screening colonoscopy is generally reimbursed for persons over the age of 55 years [4] with an annual uptake of about 3 % [5]. Although colonoscopy is generally open to all insured persons 55 years or older, no invitation or reminder system exists in Germany and uptake depends on the initiative and interest of general practitioners, gynaecologists, urologists and other specialists, who refer patients interested in CRC screening to gastroenterologists. A variety of media campaigns [6] and other initiatives have led to only small and brief bursts of interest. The reasons for the limited take-up of CRC screening, especially of colonoscopy, are diverse and not fully known; some recent studies have looked into the issue of barriers to CRC screening in general, the acceptability of various tests and potential measures to improve participation [7-16]. Apart from general doubts and fears, factors such as perception of faecal occult blood testing (FOBT) as unpleasant and colonoscopy as painful may have contributed to the lack of uptake.

Capsule endoscopy was introduced some years ago primarily for small bowel diagnostics, but has been extended to the colon with a modified capsule used for capsule colonoscopy. Capsule colonoscopy has been shown to be about 65 %-75 % accurate for adenoma detection in the large bowel when compared with colonoscopy, with better results for a more recent colon capsule version [17-20]. It could be speculated that the use of this new technology, although still requiring bowel cleaning, might attract more people potentially interested in CRC screening.

Therefore, the present prospective study evaluated the uptake of capsule as an alternative to conventional colonoscopy when offered to insured persons in the framework of invited CRC screening.

## Patients and methods

### Study partners

The study was designed and supervised by the Department of Interdisciplinary Endoscopy, University Hospital Hamburg-Eppendorf (S.G., TR), and executed by four local gastroenterologists in private practice (R.B., H.H., M.B., M.B.), supported by a hospital-based gastroenterologist (H.K.) with previous experience in capsule colonoscopy, in the area of Rinteln, Hameln and Wunsdorf, Lower Saxony, Germany, in close cooperation with a regional medical insurance company (BKK24). This area south of Hannover has a population of 1 307 568 (source: http://www.meinestadt.de), and BKK 24 has 14 304 insured persons in this area, 3091 of whom were above the age of 55 in 2009. About 75 % of all persons insured with BKK 24 in Lower Saxony were living in the study area (see below). The study was approved by the IMDEC GmbH Ethical Committee, Freiburg (31.3.2009).

### CRC uptake in the Rinteln area

In Germany, since the end of 2002, persons above the age of 55 who have not undergone colonoscopy in the preceding 10 years are entitled to a screening colonoscopy (which can be repeated after 10 years if negative), without an existing invitation or reminder programme. The nationwide current annual uptake of screening colonoscopy is around 3 % [5]. According to BKK 24 data for the Rinteln area, the uptake of screening colonoscopy in Lower Saxony among their insured persons above the age of 55 has been 1.02 % for the last 3 years before the start of the study, and was set at 1 % for study purposes. This uptake is lower than on National average in Germany according to data from the National screening colonoscopy registry [6].

### Organisation of the present study

At the beginning of the study an introductory meeting was held to guarantee acceptance by all the medical partners in the area, and for discussion and agreement on similar consultation procedures (a summary of bullet points was provided for the informed consent) for the persons showing an interest who responded to the BKK 24 invitation letters. Balanced informed consent about pros and cons of colonoscopy versus capsule endoscopy was agreed upon. The 4 gastroenterologists underwent special training in performance and reading of capsule colonoscopy. Invitation letters were sent out by the BKK 24 medical insurance company to a total of 2150 eligible persons in the area of Rinteln and surrounding cities, with about 25 % each invited in the spring and summer of 2009 and about 50 % in the autumn, starting in Rinteln (where the participating gastroenterologists were located) and subsequently involving people living but within 50 km.

Persons opting to undergo colonoscopy gave informed consent and underwent standard lavage regimens for colon preparation. Those willing to undergo capsule colonoscopy (Pillcam Colon 1, Given Imaging Corp. Hamburg/Germany) were provided with a detailed information sheet about capsule accuracy and the need for repeat colonoscopy if positive (including preparation) as well as about capsule-specific bowel preparation, as described fully elsewhere [17,18]. If polyps were found, capsule patients were re-invited for colonoscopy. In case of insufficient bowel preparation for adequate capsule reading or incomplete passage of the large bowel, patients were also offered colonoscopy. Study participants were given questionnaires before and after an initial meeting with the gastroenterologist, with items about their motivation and their decision for either capsule or conventional colonoscopy. The non-responders could not be contacted for their motivation not to participate for data safety reasons.

### Outcome parameters

*The main outcome parameter* was the potential increase in the rate of persons accepting conventional or capsule colonoscopy among all persons invited compared with the mean annual uptake of colonoscopy in the preceding 3 years (1 %, see above).

*Secondary outcome parameters* were

· Adenoma yield in both groups, namely the capsule group including those participants with subsequent colonoscopy, and the group with capsule colonoscopy only

· Rate of capsule examinations with sufficient bowel preparation (grading was done on a 4-point scale in accordance with previous studies [17,18]

· Adverse events and complications in both arms of the study

· Patient opinion and acceptability according to the questionnaires mentioned above

### Statistical analysis

No precise case number calculation could be made since this was the first study of its kind focusing on uptake when an alternative to colonoscopy was available. The current annual acceptance rate for screening colonoscopy in Germany is around 3 %, in the Rinteln area it is 2.2 %, and among persons insured with BKK 24 it is around 1 % (see above). Thus, a significant difference in uptake, for a one-sided comparison, would be reached at an increased uptake of 1.6 % versus 1 % (p = 0.045), and for a two-sided comparison at 1.7 % (p = 0.049).

## Results

### General acceptance and uptake

**Figure 1:**
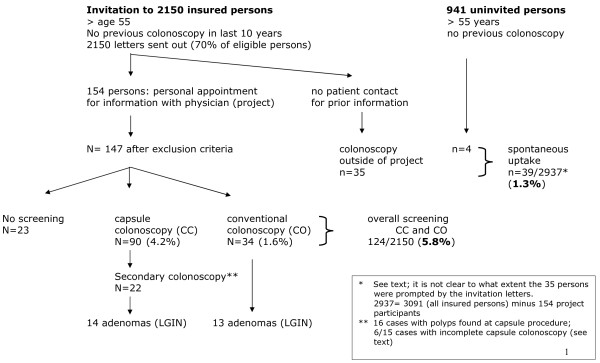
Flow sheet of study procedure and outcome of participants and other screening colonoscopies in 2009.

Up to December 31st 2009, 2150 letters (49.3 % to men, and 51.7 % to women) had been mailed to insured persons insured with BKK 24 in the Rinteln area who were over 55 years of age and eligible for CRC screening in Lower Saxony. Of those, 154 persons (88 men, 66 women, mean age 63.5 years, SD 6.2) contacted one of the 4 gastroenterologists named in the letter and presented for a personal interview. After this discussion and reading the standardized informed consent, 7 were excluded since they were not eligible for screening, and 124 of the remaining 147 persons decided to undergo either colonoscopy (n = 34) or capsule (n = 90), while 23 finally opted against both forms of endoscopic screening. Details are shown in Figure 1. The mean age of the screenees was 63.3 years in the colonoscopy group and 62.7 in the capsule endoscopy group; the percentage of men was higher in the capsule group (64.4 % vs. 47.1 % for colonoscopy).

There were 39 persons undergoing screening colonoscopy outside of the project during the study period who were insured with BKK 24. 35 of those had received the invitation letters but did not seek further information as offered within the project; for these people it is not clear whether they were prompted by the invitation letter since IRB-based data protection did not allow for identification of individuals and a timewise correlation of invitation letter and colonoscopy. It was decided to count these 39 persons as the spontaneous uptake group which would correspond to a figure of 1.3 % instead of 1 % observed in the years before (which was set as background uptake).

Thus, the uptake of any endoscopic screening test (capsule or conventional colonoscopy) stimulated by the project invitation letters was 5.8 % (124/2150), with 34 (1.6 %) opting for primary colonoscopy and 90 (4.2 %) choosing primary capsule endoscopy. Comparing these rates to the spontaneous rate set at 1 %, the increase was 60 % for colonoscopy (1.6 % versus 1 %, p = 0.075; two sided comparison) and more than 4 fold - 420 % (4.2 % versus 1 %; p < 0.001) – for capsule colonoscopy. Regarding sex distribution, uptake was 5.6 % in men and 2.8 % in women (p = 0.002).

### Reading and bowel cleanliness in the capsule group

Bowel cleanliness was graded excellent in none, and good in 28, moderate in 45 and poor in 11 cases (12.2 %). Incomplete colonic capsule passage was found in 15/89 cases, and in 7 of those the rectum was reached. In one case spontaneous passage through the colon occurred before the capsule was activated. Thus, full colonic visualization on the basis of complete capsule passage was possible in 73/89 cases (82 %).

### Adenoma yield

These data are shown in Figure 1. Overall, 9 patients with 13 low-grade adenomas (all <1 cm) were found in the *colonoscopy group*. This accounts for a patient rate with at least one adenoma of 26.4 % (confidence limits [CL] 12.9 %, 44.4 %) and an adenoma rate (all adenomas/all patients) of 38.2 % (CL 22.2 %, 56.4 %); this difference was statistically significant (p = 0.018, Fisher exact tests, two sided). Adenomas in the *capsule group* were only counted if confirmed by colonoscopy with biopsy or polypectomy. In this group, 16 patients underwent colonoscopy because of positive capsule findings suggesting polyps; in addition, 6/15 with incomplete capsule endoscopy followed the recommendation to undergo colonoscopy. Of these 22 patients overall, 8 cases were identified who had a total of 14 adenomas (of those, 5 were 1 cm or greater) in the capsule group. In summary, the patient rate with at least one adenoma for capsule colonoscopy, based on secondary colonoscopy results, was 9 % (8/90; CL 4.7 %, 18.1 %), and the adenoma rate (all adenomas/all cases) was 15.5 % (CL 8.8 %, 24.7 %). Also this difference was statistically significant (p = 0.013, Fisher exact tests, two sided)

### Adverse events

There were no reported adverse events in any of the study participants.

### Patient opinion and acceptability

147 persons undergoing capsule and/or conventional colonoscopy within the project responded to the questionnaires (Table 1). The interest in endoscopic CRC screening was aroused by the BKK invitation (85.5 %), followed by recommendation from a general physician (19.7 %). Amongst those persons who eventually underwent one of the two tests, the main reason for a final choice of capsule was the fear of colonoscopy-related discomfort and complications, while the main reason for choosing colonoscopy was the possibility for taking tissue samples and carrying out polypectomy.

**Table 1:** Results of patient questionnaire; multiple positive responses were possible for each item

Reasons for general interest in colorectal cancer screening (n = 147)*		
I think prevention is generally important	111	75.5 %
My general physician recommended screening	29	19.7 %
Mainly due to the invitation by BKK 24	125	85.0 %
Since capsule colonoscopy is free	52	35.4 %
Others	8	5.4 %
Reasons to choose capsule colonoscopy (n = 89)**		
Sounds more pleasant	82	92.1 %
I am afraid of colonoscopy pain	10	11.2 %
I am afraid of sedation	4	4.5 %
I am afraid of colonoscopy complications	11	12.4 %
Others	2	2.2 %
Reasons to choose colonoscopy (n = 37)**		
Colonoscopy is the standard method	8	21.6 %
Colonoscopy enables biopsy and polypectomy	31	83.8 %
Others	2	5.4 %

*from questionnaire before the interview with physician.

**from questionnaire after the interview with physician.

The acceptability of the different procedures as perceived after the examinations was investigated. Among participants in the capsule group who were asked whether capsule colonoscopy would be again the method of choice for repeated colonic examination, 65 % answered “yes” and 22 % “probably yes”, after capsule colonoscopy. The corresponding values for colonoscopy were 94 % and 0 %. Of the 22 persons who underwent colonoscopy after capsule, 16 answered the questionnaire and 11 stated they would choose conventional colonoscopy for a repeat examination, mainly because everything could be done in one procedure and colonoscopy was felt to be more accurate. Two further persons said that they would probably choose capsule rather than conventional colonoscopy.

## Discussions

The present study for the first time analyses the effect of offering a new examination method on the uptake of CRC screening. In Germany, CRC screening by colonoscopy is based on spontaneous uptake and no invitation system exists as yet. In our study we were able to show that in addition to the presumed invitation effect – which per se led to an increase in colonoscopy rate by about 60 % – a fourfold increase in endoscopic screening could be attributed to the offer of capsule colonoscopy, with men in particular finding capsule colonoscopy more acceptable. However, the overall adenoma yield was not different in the two examination groups, although all patients with positive capsule findings and 40 % with incomplete capsule examination underwent secondary conventional colonoscopy. This points towards a need to improve capsule colonoscopy diagnostic efficacy before it can be considered for implementation into a CRC screening programme.

Our study results deserve several comments which have only partly to do with limitations of the present study:

1. Uptake was rather low even under study conditions using an invitation process; however, uptake within the German opportunistic screening programme is generally limited (around 3 % annually) but was lower in the study area in the years preceding the study (1 %). It could be that the effect of offering capsule endoscopy may be less pronounced in areas with higher uptake, but this would have to be studied.

2. We performed a single-arm rather than a randomized study; from a purely scientific standpoint, only the latter, randomizing patients to being invited to either colonoscopy or capsule endoscopy would have enabled us to clearly differentiate between the invitation effect and the effect of offering a new technique. Nevertheless, our design mirrored the reality where often more than one options are offered. A further limitation to generalize our result was that only one patient group covered by one insurance was included in our study.

3. In the literature, direct comparisons of spontaneous versus invited screening colonoscopy uptake rates are not available, since it is difficult to measure the spontaneous uptake outside of a programme. However, there is ample indirect evidence that invitation methods increase uptake, and there are also randomized and other studies showing that more intensive invitation measures and the involvement of general practitioners in the invitation process lead to greater uptake [21-25]. In our study, for reasons of data protection, 35 persons who underwent colonoscopy and who also had received invitation letters, but did not enter the project and/or sought information from one of the 4 project gastroenterologists, were not counted as study participants since their individual data could not be used. We decided that these cases would not be counted among the persons with concomitant spontaneous uptake. If we did this, the uptake increase would have been much higher for colonoscopy, further underlining the importance of an invitation process. Naturally, screening behavior would furthermore be different in setting with free access to the respective screening test versus self-paid methods; since colonoscopy is reimbursed over the age of 55 (without colonoscopy in the preceeding 10 years), we think that free access to capsule colonoscopy allowed for a fair comparison in the German setting. Very likely, this would change with changing reimbursement strategies.

4. Data on adenoma yield by capsule colonoscopy was disappointing, although our study was not powered to show differences in polyp yield. Firstly, it was noteworthy that all persons with polyps detected on capsule endoscopy also agreed to undergo secondary colonoscopy; in the group with incomplete capsule endoscopy, this rate was lower but still relevant (40 %). In the recent large multicenter trial on capsule endoscopy controlled by conventional colonoscopy, sensitivity for polyps less than 1 cm was only slightly over 60 % [19]. Thus, it is conceivable that under routine conditions as in our study, sensitivity can be even lower; we think that sensitivity would have to be substantially improved before capsule colonoscopy would be an option for CRC screening. Polyps also tended to be smaller in the colonoscopy group which might be related to the lower sensitivity of capsule for smaller polyps [19]. It appears possible that this would be the case with the second generation colon capsules [20,26], but this would have to be proven in either a similar study and/or a comparative trial

5. The offer of capsule endoscopy had a much better effect on uptake in men than in women. Men are known to have a lower uptake than women of colorectal screening measures, at least in Germany [27]: a detailed analysis of colonoscopy screening participation in Germany shows a 10 % lower uptake of screening colonoscopy by men, which is increased in the age groups below 70 [5]. Experiences from other countries are however variable and more difficult to predict [28,29]. Our results show that men might be better motivated by the offer of this new technology, but these results are of course preliminary. The durability and reproducibility of such effects at repeated investigation is not known either.

In our study, a ‘one-stop shop’ process – using one colonic preparation for capsule endoscopy, followed by colonoscopy in case of lesions, requiring very rapid reading - was not attempted or done (and this was explained to patients during informed consent). Patients with positive or doubtful findings therefore had to undergo a second bowel preparation. This may be regarded as drawback of capsule endoscopy, even if it only applies to 20 %–30 % of patients. In previous capsule studies where colonoscopy was used as the gold standard in all cases, such as in the large multicenter trial [19], the protocol included a single preparation for capsule plus colonoscopy procedures, but this did not include patient selection for colonoscopy by capsule as a filter test when positive, since capsule results were not known at the time of colonoscopy. It may be quite difficult to establish such programmes of capsule endoscopy, with performance and reading, plus colonoscopy, all routinely done in 1 day. Further studies have to show how such ambitious programmes might possibly be implemented, for example by fast central reading services and an on-call colonoscopy service for screenees with polyps identified by the capsule, and whether such programmes might further increase uptake. Only if larger and stable numbers of capsule colonoscopies should ever be performed, and regarded as economically viable, might such a service appear more realistic. It will then also become more evident to which degree uptake is influenced by other factors such as capsule performance (sensitivity, need to still undergo colonoscopy) and logistical issues (such as one-stop shopping).

## Conclusions

The present study showed that uptake of colorectal screening can be increased by offering capsule endoscopy in addition to colonoscopy. These interesting new results with regards acceptance as well as performance of capsule colonoscopy as a screening option in daily routine can form the basis of further studies. In particular, capsules with increased sensitivity such as those from the second generation should be used for further larger and preferentially randomized studies to match uptake with outcome with respect to adenoma detection.

## Competing interest and funding

There is no conflict of interest or any financial involvement of any of the medical authors. Given Imaging Ltd. (Hamburg/Germany for the European Branch) provided the capsules for the study at a reduced price and helped in study organisation; Given imaging also covered the publication charges. BKK-24 Medical Insurance (CEO Friedrich Schütte is co-author) supported the study by financing the project with a special contract on integrated patient care (§§ 140a ff. SGB V) and by sending out invitation letters to potential screenees.

Furthermore, the corresponding author had full access to all of the data and takes full responsibility for the veracity of the data and statistical analysis.

## Authors’ contributions

SG was involved with study planning, performance, audit, data collection and paper writing, HK, RB, HH, MB and MB were involved in study performance and patient care, NP helped with data collection and monitoring, FS (CEO of BKK 24 medical insurance) was involved in study planning and organization (writing invitation letters to possible screenees), UG is responsible for study statistics, JFR was involved in study planning and paper correction, LA provided the comparative data from the German National Screening Colonoscopy Registry and Thomas Rösch (responsible author and principal investigator) was responsible for study planning and organisation, data monitoring and analysis and paper writing. All authors read and approved the final manuscript.

## Pre-publication history

The pre-publication history for this paper can be accessed here:

http://www.biomedcentral.com/1471-230X/12/80/prepub
